# Naturally-occurring changes in social-cognitive factors modify change in physical activity during early adolescence

**DOI:** 10.1371/journal.pone.0172040

**Published:** 2017-02-10

**Authors:** Rod K. Dishman, Marsha Dowda, Kerry L. McIver, Ruth P. Saunders, Russell R. Pate

**Affiliations:** 1 Department of Kinesiology, University of Georgia, Athens, GA, United States of America; 2 Department of Exercise Science, University of South Carolina, Columbia, SC, United States of America; 3 Department of Health Promotion, Education, and Behavior, University of South Carolina, Columbia, SC, United States of America; Vanderbilt University, UNITED STATES

## Abstract

**Purpose:**

To determine whether naturally-occurring changes in children’s motives and beliefs are associated with the steep decline in physical activity observed from childhood to early adolescence.

**Methods:**

Latent growth modeling was applied in longitudinal tests of social-cognitive influences, and their interactions, on physical activity in a large cohort of boys and girls evaluated annually between 5^th^ and 7^th^ grades.

**Results:**

Measurement equivalence of motives and beliefs was confirmed between boys and girls. After adjustment for gender and maturity differences, physical activity declined less in children who reported the least decreases in self-efficacy for overcoming barriers to activity and perceived parental support. Physical activity also declined less in students who persistently felt they had more parental and friend support for activity compared to those who reported the largest decrease in support from friends. After further adjustment for race, the decline in physical activity was less in those who had the largest decrease in perceived barriers and maintained a favorable perception of their neighborhood environment. Changes in enjoyment and social motives were unrelated to change in physical activity.

**Conclusion:**

Using an objective measure of physical activity, we confirm that naturally-occurring changes in children’s beliefs about barriers to physical activity and their ability to overcome them, as well as perceptions of their neighborhood environment and social support, are concurrent with age-related declines in children’s physical activity. The longitudinal findings confirm these putative social-cognitive mediators as plausible, interacting targets of interventions designed to mitigate the marked decline in physical activity that occurs during the transition between elementary and middle schools.

## Introduction

Physical inactivity is regarded worldwide as a public health burden during adolescence, which is a foundational period for future health [1]. Physical activity among U.S. youths is below recommended levels [2, 3], and it decreases steeply in children and youths between ages 9 to 15 years in the U.S. [4, 5] and in other nations [6–8]. This age-related decline in physical activity is thought to be a major contributor to the increased rates of overweight and obesity observed during this developmental period [9]. Accordingly, many authorities have recommended that public health interventions be launched to promote higher levels of physical activity in young people [10, 11]. An important goal of such interventions is to reduce the pronounced age-related decline in physical activity during the transition from childhood to early adolescence.

Interventions to increase physical activity in children and youths have achieved only modest success, on the whole, in sustaining meaningful change [12–16]. The Physical Activity Guidelines for Americans Midcourse Report[12] and others [17] recommended that long-term longitudinal assessment of youth physical activity, which is the focus of the study we report here, is a high priority for future research. We measured physical activity using accelerometry, which is a preferred method for the objective measurement of physical activity in children and youths [18].

One explanation for the limited success of past physical activity interventions is that most of them did not target or alter child-level factors that are putative mediators (i.e., causal explanations) of physical activity change (e.g., children’s motives and beliefs that theoretically transmit or modify an intervention effect) [19]. This has mainly resulted from a dearth of evidence confirming that change in these presumptive determinants of physical activity in children and adolescents is, in fact, prospectively associated with measured change in physical activity [20]. Of equal importance, it is not known how these candidate mediators of physical activity interact to moderate each other’s influence on change in physical activity. Furthermore, previous studies have not examined how change in physical activity and its mediating or moderating influences by children’s beliefs and motives depend on maturity, which accelerates during early adolescence. Prior evidence suggests that earlier maturation partly accounts for lower physical activity in girls compared to boys [21–24] and, hence, might obscure the relationship of physical activity with change in social-cognitive factors that can also be influenced by maturity during early adolescence [25].

In this study, we built on our prior studies of modifiable motives (i.e., social and enjoyment) and beliefs (i.e., self-efficacy and perceptions of barriers, support from parents and friends, and the perceived neighborhood environment) about physical activity among adolescent girls [26–29] by observing prospective associations of these social-cognitive variables with change in objectively measured physical activity in a cohort of boys and girls as they transitioned from elementary school to middle school. We examined direct and moderated (i.e., interactions) effects of change in the variables on age-related decline in physical activity, while adjusting for maturation. Although motives and beliefs are formed in large part by social learning and self-appraisal as well as reinforcement history [30], whether they change naturally during the transition from elementary to middle school had not been determined by prior evidence. Based on theory elaborated in our prior studies [26–29] and elsewhere [19], we hypothesized that changes in the social-cognitive variables, and their interactions, would be related to change in physical activity.

## Methods

### Study design

The study was approved by the Institutional Review Board (IRB) of the University of South Carolina (approval #PRO00003730). The Transitions and Activity Changes in Kids (TRACK) study employed a prospective cohort design. Students and their parents were enrolled in the study when the children were 5^th^ graders. A data collection protocol was administered when the students were in the 5^th^, 6^th^ and 7^th^ grades, thereby providing observations across a period that spanned the transition from elementary to middle school. Consistent with the Social Ecological Model [31], the overarching goal of TRACK was to identify factors ranging from intra-personal to physical environmental that influence change in children’s physical activity levels during a critical developmental period. The overall study protocol included collection of data from the children, their parents, and school personnel, as well as characterization of the physical environment of the communities in which the families resided. The analyses presented here are based on information provided by the children, and latent growth modeling procedures were used to identify changing factors that were related to changes in physical activity levels across the two-year observation period.

### Participants

The multi-ethnic sample included 857 5^th^ grade boys and girls recruited in Spring and Fall 2010 from elementary schools located in two school districts in South Carolina, as described in detail elsewhere [32]. Fourteen of the 17 elementary schools and subsequently all seven middle schools in one district and all seven elementary and all six middle schools in the other district agreed to participate. Active consent and assent forms approved by the IRB were sent home with students, and written forms completed by parents or legal guardians were returned to the schools. The race/ethnicity proportions were approximately: 36% non-Hispanic black, 37% non-Hispanic white, 9.5% Hispanic/Latino, 3% Asian/Pacific Islander, 3% American Indian, and 11% multi-racial. The samples were not randomly selected, but their gender and race/ethnicity were generally representative of the school populations. The median (low to high quartiles) percentage of students below the Federal poverty level was 14.7% (11.1% to 19.3%). Fifty-seven percent of parents had attended or graduated from college or technical school, another 11% had attended or graduated from graduate school, and seven percent had not completed high school. Body Mass Index (BMI, kg/m^2^) (mean ± SD) was higher in girls (p< .001); 46–49% of girls and 43–46% of boys had a BMI ≥ 85^th^ percentile based on sex-specific BMI-for-age growth charts published by the Centers for Disease Control and Prevention (CDC) [33]. Table 1 presents participant characteristics according to gender.

**Table 1:** Participant characteristics according to gender.

	5^th^ Grade		6^th^ Grade		7^th^ Grade	
	Males	Females	Males	Females	Males	Females
	(n = 397)	(n = 460)	(n = 396)	(n = 456)	(n = 362)	(n = 430)
Age, years	10.6 (0.5)	10.6 (0.5)	11.5 (0.5)	11.5 (0.5)	12.5 (0.5)	12.5 (0.5)
BMI, kg/m^2^	20.7 (4.8)	21.6(5.0)	21.3 (5.0)	22.3 (5.4)	21.9 (5.2)	23.2 (5.7)
Race, %						
Non-Hispanic Black	38.8	34.1	38.9	34.0	39.8	34.7
Non- Hispanic White	35.2	39.1	35.4	39.3	35.4	39.3
Hispanic/Latino	9.1	9.8	9.1	9.6	8.7	9.8
Asian/Pacific Islander	3.3	2.0	3.0	2.0	3.3	1.9
American Indian	4.5	2.2	4.5	2.2	4.1	2.0
Multi-racial/Other	9.1	12.8	9.1	12.9	8.7	12.3
Maturity offset, years	-2.37 (0.57)	-0.55(0.71)	-1.70 (0.65)	0.20 (0.65)	-0.85 (0.74)	0.98 (0.59)
Poverty, %	16.2 (6.7)	16.3 (7.4)	16.3 (6.9)	16.2 (7.2)	16.0 (6.9)	16.3 (7.3)
Parent Education, %						
Attended high school	6.0	7.6	5.2	8.7	5.5	9.6
Completed high school	21.6	27.7	21.6	25.3	19.9	25.8
Attended college	34.5	27.4	33.2	26.1	30.3	24.6
Completed college	26.7	26.3	29.3	28.6	33.2	28.4
Attended grad school	2.0	3.8	3.0	4.9	2.2	3.8
Completed grad school	9.2	7.2	7.6	6.1	8.9	7.8

### Data collection procedures

Data collection procedures were carried out at each school according to a manual of procedures by a trained measurement team over two visits with each participant. During the first visit, participants completed the questionnaires by entering their responses into a survey software database on laptop computers, had anthropometric measurements taken, and received an accelerometer. Participants completed the measures in small groups (≤ 24 students), at times and places determined by each school. Participants returned the accelerometer at the second visit.

#### Anthropometric variables

Height and weight were each assessed with two trials using a Seca height board and a Seca Model 880 weight scale. Standing and seated height measurements were repeated and averaged if the difference between the two measurements was ≥ 0.5 cm. Weight measurements were repeated if the difference was ≥ 0.5 kg. BMI was expressed as kg/m^2^ and as standardized scores (BMz) based on CDC growth charts [34]. Maturity was assessed by years from estimated peak height velocity using a longitudinally validated prediction equation for boys and girls [34, 35]. Each student responded to two questions about race/ethnicity. The first asked whether the student thought of himself or herself as Hispanic or Latino. The second asked about student’s self-identification as American Indian or Alaskan Native, Black/African American, Native Hawaiian or other Pacific Islander, White, Asian or other (e.g., multi-racial).

#### Socio-economic variables

Parents reported their highest level of education (1 = attended high school, 2 = completed high school, 3 = attended college or technical school, 4 = completed college or technical school, 5 = attended graduate school, 6 = completed graduate school). Percent poverty was calculated using the US Census American Community Survey variable “Poverty status in the past 12 months” based on the Census tract of each child’s place of residence [36].

#### Physical activity

Each child wore an Actigraph accelerometer (models GT1M and GT3X, Pensacola, FL) during waking hours for 7 consecutive days, except while bathing, swimming or sleeping. Accelerometer counts in the vertical plane were collected and stored in 60-sec epochs and reduced using methods previously described [36]. Total physical activity (light + moderate + vigorous physical activity (PA) was expressed as mean daily minutes per hour (min/hour) of wear time. Data for Sunday were excluded from analysis because of poor wear rates and low reliability. Eighty percent of children provided accelerometer data for eight or more hours of daily wear on four or more days, representing 77% of the total records possible on Monday through Saturday. Missing values for children with at least two days of 8 or more hours of wear each day were estimated using Proc MI in SAS (Version 9.3, SAS Institute, Inc., Cary, NC). There was a quadratic drop in wear time (mean ± SD) of 0.7 hours (95% CI = 0.5 to 0.9) from 5^th^ grade (12.5 ± 0.9 hours) to 6^th^ grade (11.8 ± 1.0) and 0.2 hours (95% CI = 0.18 to 0.22) to 7^th^ grade (11.6 ± 1.0), *P*< .001, that differed by elementary school, *P* = .012, so physical activity was expressed as min/hour of wear time. Reliability across the 6 days in the samples reported here was 0.84 in 5^th^ grade, 0.88 in 6^th^ grade and 0.88 in 7^th^ grade. Stability coefficients were .64, .61, and .52 between 5^th^ and 6^th^ grades, 6^th^ and 7^th^ grades and 5^th^ and 7^th^ grades, respectively. Physical activity data were available for a cohort of 857 children assessed in 5^th^ grade and in either or both of 6^th^ (N = 778) or 7^th^ (N = 686) grades.

#### Social-cognitive variables

A student questionnaire was developed for the purpose of measuring the putative mediators and moderators of change in physical activity consistent with prior validation studies of 5^th^ grade boys and girls and 6^th^ grade girls [37] and 6^th^ and 8^th^ grade girls[27, 38–40]. The questionnaire with evidence for the validity of the scales is available elsewhere [32, 41]. Unless otherwise noted, a four-point ordered scale format was used. Results are reported as mean scores per item for each scale. Missing responses to items on the questionnaires ranged from 0% to 3%.

#### Self-efficacy

Efficacy beliefs about overcoming barriers to physical activity was measured using 8 items developed for use with 5^th^ grade boys and girls and re-specified for use with 6^th^ and 8^th^ grade girls [38, 39–42].

#### Perceived barriers

The 9-item version of a measure developed for the Trial of Activity in Adolescent Girls (TAAG) study was used. It is comprised of three 3-item scales for assessing obstacles, evaluation, and outcomes as barriers to physical activity that are subordinate to a single secondary factor [41].

#### Enjoyment motivation and social motivation for physical activity

Two Likert-type scales assessed presumably intrinsic (i.e., Enjoyment; 7 items) and extrinsic (i.e., Social; 5 items) incentives for participation in physical activities [43], consistent with self-determination theory [44] and modified for reading level [38].

#### Perceived neighborhood environment

The children were asked to respond on a 5-point Likert-type scale to 12 items derived from previously validated questionnaires.[45]

#### Perceived parent support

Four items from the student survey of the Amherst Health and Activity Study [46], previously validated for use in adolescent girls, were used to assess children’s perception of support for physical activity provided by parents or guardians [42].

#### Perceived friend support

Three items from the student survey of the Amherst Health and Activity Study,[46] previously validated for use in adolescent girls [27], were used to assess children’s perception of support for physical activity provided by friends.

### Statistical analysis

#### Latent growth modeling

Trajectories of change in physical activity and the putative mediators or moderators were estimated using latent growth modeling in Mplus 7.4 [47] with robust maximum likelihood estimation of parameters with full information imputation, which is robust with up to 25% missing data [48]. Multi-level models were used to estimate between-school differences in physical activity and the putative mediators and moderators [47]. Adjustment was made for nesting effects of students within schools by correcting the standard errors of parameter estimates for between-school variance using the Huber-White sandwich estimator, which is robust to heteroscedasticity and group-correlated responses [47]. Parameters and their standard errors were estimated for initial status (i.e., mean at 5^th^-grade baseline), change (i.e., slope of differences across the 3 time points from 5^th^ to 7^th^ grades), and the variances (i.e., inter-individual differences) of initial status and change. Critical z-scores (parameter estimate/SE) were used to test significance.

**Fig 1:**
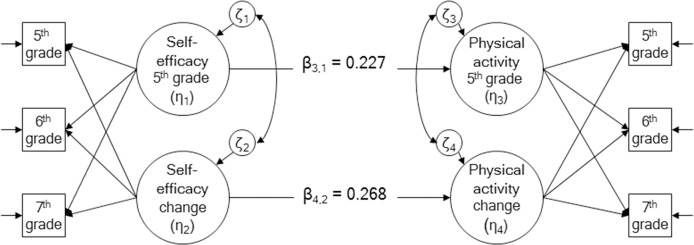
Growth model.

Prediction of change in physical activity was tested by including initial status and change in physical activity regressed on initial status and change in each social-cognitive mediator, while setting the regression of change in the mediator on initial status at zero (see Fig 1 for self-efficacy as an example). Regression coefficients were compared between boys and girls in a multi-group model using the Wald test. Interactions between changes in social-cognitive variables (or between change in a variable with 5^th^ grade level in another variable that did not change) were tested using standard procedures [47, 49].

To account for effects of gender, race/ethnicity (black, Hispanic, and all others vs. white or vs. black), parental education, and poverty level on change, those variables were added to the LGM models as covariates. Maturity was tested as a time-varying covariate. Growth models (initial values in 5^th^ grade and change between 5^th^ and 7^th^ grades) were otherwise compared between boys and girls in a multi-group model using on the Wald test. The longitudinal measurement equivalence/ invariance (MEI) of the model for each scale was examined between boys and girls by testing the equality of item variance and covariance between boys and girls across the three measurement periods [50].

Model fit was evaluated with multiple indices. The chi-square statistic assessed absolute fit of the model to the data. However, the test is almost always significant when samples approximate or exceed 400 cases, so other fit indices were also used, as recommended elsewhere [51]. Values of the Comparative Fit Index (CFI) ≥ 0.90 and 0.95 were used to indicate acceptable and good fit. Values ≤0.06 of the root mean square error of approximation (RMSEA) and the standardized root mean square residual (SRMR) were used to represent close fit. Values ≥ 0.96 for CFI in combination with values of the SRMR ≤ 0.10 results in the least sum of type I and type II error rates [51]. The sample size was adequate for model tests. Statistical power exceeded .90 at an alpha of .05 for rejecting good fit at a RMSEA of .06 and a conservative estimate of model complexity at 10 df [52, 53]. Model fit was acceptable for all growth models reported here (CFI ≥ 0.960, RMSEA ≤ 0.051, SRMR ≤ .052).

## Results

### Between-school variance

Multi-level analysis indicated small and non-significant variance between schools (intra-class correlation coefficient; ICC) in physical activity in 5th (.058, p = .076), 6^th^ (.025, p = .233), and 7^th^ (.027, p = .140) grades. Between-school variance in the social-cognitive variables was also small (ICC ≤ .048) and was significant only for perceived environment in 6^th^ grade (p = .049). Between-school variance in maturity was similarly small (ICC ≤ .045) and reached significance only in 5^th^ grade (p = .019). Hence, tests of cross-level influences of school features on child-level associations between those variables were not conducted. Nonetheless, elementary school was retained as a cluster variable in all models to provide precise and conservative parameter estimates to adjust for nesting effects within schools [47].

### Measurement equivalence/invariance

Longitudinal tests of equal variance and covariance of scale items between boys and girls across the three years between 5^th^ and 7^th^ grades supported MEI for all social-cognitive scales (CFI ≥ 0.955, RMSEA ≤ 0.071, SRMR ≤.100). Table 2 shows tests and fit indices for each measurement scale.

**Table 2:** Longitudinal tests of measurement equivalence/invariance (equal variance and covariance) between boys and girls across 5^th^ through 7^th^ grades.

Scale	χ^2^	df	p-value	CFI	RMSEA (90% CI)	SRMR
**Self-efficacy**	255.8	180	< .001	0.980	0.031 (0.022–0.040)	0.079
**Perceived barriers**	135.0	75	< .001	0.955	0.028 (0.000–0.044)	0.100
**Motives**						
**Enjoyment**	66.3	50	.061	0.993	0.028 (0.000–0.044)	0.100
**Social**	61.4	30	< .001	0.968	0.049 (0.032–0.067)	0.066
**Perceived environment**	320.6	225	< .001	0.983	0.031 (0.023–0.039)	0.043
**Perceived parental support**	63.7	50	.092	0.996	0.026 (0.000–0.044)	0.049
**Perceived friend support**	94.3	30	. < .001	0.970	0.071 (0.055–0.087)	0.073

### Growth models

#### Physical activity

**Fig 2:**
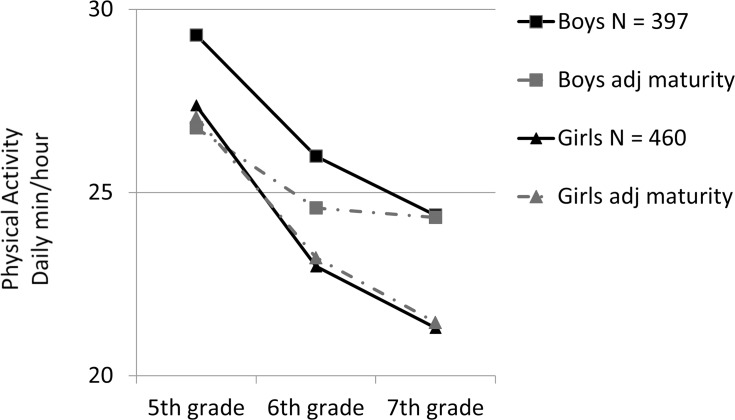
Change in physical activity.

Boys and girls differed on initial values and change trajectories (p < .001). See Fig 2. Mean physical activity in the 5^th^ grade was 29.3 min/hour (95% CI = 28.4 to 30.2) in boys and 27.37 min/hour (95% CI = 26.8 to 27.9) in girls. There was a decline from 5^th^ grade to 6^th^ grade for boys (-3.31 min/hour (95% CI = -4.14 to -2.47, *P* < .001)) and girls (-4.39 min/hour (95% CI = -4.99 to -3.79) *P <* .001)). Thereafter, negatively accelerated declines were observed between 6^th^ and 7^th^ grades of -1.61 min/hour (95% CI = -1.84 to -1.38) for boys and -1.68 min/hour (95% CI = -1.82 to -1.54) for girls. Based on wear time, physical activity dropped 10 min/day (95% CI = -11.2 to -8.8) for boys and 11.8 min/day (95% CI = -13.2 to -10.4) for girls between 5^th^ to 6^th^ grades; drops of 16% and 21%.

#### Maturity

Boys and girls also differed on initial peak height velocity offset (< .001) and change trajectories (p < .002). Mean peak height velocity offset in the 5^th^ grade was -2.38 years (95% CI = -2.46 to -2.29) in boys and -0.56 years (95% CI = -0.67 to -0.44) in girls. The decrease was 0.69 (95% CI = 0.64 to 0.74) years in boys and 0.75 (95% CI = 0.71 to 0.79) years in girls between 5^th^ and 6^th^ grades and 1.26 (95% CI = 0.90 to 1.62) years in boys and 0.87 (95% CI = 0.71 to 1.02) years in girls between 6^th^ and 7^th^ grades.

Physical activity levels in 5^th^ grade no longer differed significantly between boys and girls (P = .770) after adjustment for maturity. Adjusted physical activity level (± 95% CI) was 26.80 (24.77 to 28.76) min/hour in boys and 27.06 (26.48 to 27.63) min/hour in girls. See Fig 2. Similarly, the difference in physical activity decline between 5^th^ and 7^th^ grades also was no longer significantly different after adjusting for maturity each year (Wald test (1) = 3.2, *P* = .073). The adjusted decline was -2.19 (-3.75 to -0.62) min/hour in boys and -3.84 (-4.44 to -.3.23) min/hour in girls between 5^th^ and 6^th^ grades and -0.26 min/hour (-0.97 to 0.45) in boys and -1.76 minutes (-1.96 to -1.56) in girls between 6^th^ and 7^th^ grades. The decline in physical activity was less in black boys and girls than other children (p< .019) but was unrelated to parental education or poverty (p≥ .912).

#### BMI

Boys and girls also differed on initial BMI (p = .007) and change trajectories (p < .002). Mean BMI (kg/m^2^) in the 5th grade was 20.65 (95% CI = 20.14 to -21.16) in boys and 21.55 (95% CI = 21.04 to 22.06) in girls. The increase in BMI (kg/m^2^) per year was 0.63 (95% CI = 0.52 to 0.75) in boys and 0.83 (95% CI = 0.72 to 0.94) in girls between 5th and 7th grades. The differences between boys and girls in physical activity at 5^th^ grade and change in physical activity between 5^th^ and 7^th^ grades remained (P-values < .005) after adjustment for BMI each year.

Boys and girls no longer differed significantly on BMI or BMIz in 5^th^ grade (*P-*values *≥* .104) or on change in BMI or BMIz (*P-*values *≥* .125) after adjustment for maturity each year, so further results for physical activity and the social-cognitive variables were adjusted for maturity each year rather than BMI. Gender was retained with maturity in the reported models in order to improve the precision of parameter estimates. Growth models were not substantively different after further adjustment for race/ethnicity and the socio-economic variables, unless otherwise noted.

#### Social-cognitive variables

There were declines (SD, P-value) each year in all social-cognitive variables: self-efficacy (- 0.41, P = .002); perceived barriers (- 0.47, P = .004); enjoyment motivation (- 0.24, P = .004); social motivation (- 0.22, P = .021); perceived neighborhood environment (- 0.17, P = .006); perceived parental support (-.19, P = .027); and perceived friend support (- 0.26, P< .001). Growth model results according to gender are shown in Table 3. Boys and girls differed on initial values (*P* ≤ = .05) for all variables except perceived neighborhood environment. Girls had a bigger decline in enjoyment motivation, while boys had a bigger decline in friend support. Only social motivation differed between boys and girls, after adjustment for maturity each year. Boys had higher adjusted levels in 5^th^ grade (2.97, 95% CI = 2.81 to 3.13) and greater decline each year (-0.11, 95% CI = -0.20 to -0.02) than did girls (2.47, 95% CI = 2.38 to 2.55) and (0.049, 95% CI = -0.20 to 0.12).

**Table 3:** Growth models for social-cognitive variables according to gender.

	Boys (N = 397)		Girls (N = 460)		Boys versus Girls			
Variable					Unadjusted		Adjusted for Maturity	
	Mean (95%CI)	Change per year	Mean (95% CI)	Change per year	Initial status(p)	Change per year (p)	Initial status (p)	Change per year (p)
Self-efficacy	3.33 (3.27, 3.39)	-0.05 (-0.08, -0.02)	3.24 (3.18, 3.30)	-0.07 (-0.10, -0.04)	.05	.35	.68	.67
Perceived barriers	1.61 (1.56, 1.66)	-0.04 (-0.07, -0.02)	1.69 (1.66, 1.72)	-0.03 (-0.05, -0.01)	.001	.446	.383	.790
Enjoyment Motivation	3.65 (3.59, 3.70)	-0.02 (-0.05, 0.01)	3.58 (3.53, 3.63)	-0.06 (-0.09, -0.03)	.048	.019	.751	.237
Social Motivation	2.69 (2.63, 2.75)	0.04 (-0.01, 0.08)	2.45 (2.38, 2.51)	0.07 (0.02, 0.12)	< .001	.298	< .001	< .001
Perceived neighborhood environment	2.88 (2.79, 2.97)	-0.04 (-0.07, -0.01)	2.86 (2.79, 2.94)	-0.03 (-0.06, 0.01)	.724	.543	.751	.976
Parent support	3.43 (3.35, 3.51)	-0.076 (-0.13, -0.03)	3.29 (3.22, 3.37)	-0.19 (-0.09, 0.05)	.018	.208	.260	.407
Friend support	3.57 (3.48, 3.66)	-0.15 (-0.20, -0.09)	3.20 (3.13, 3.27)	-0.03 (-0.08, 0.01)	< .001	.002	.452	.902

After adjustment for maturity each year and gender, the decline in physical activity was related to decline in self-efficacy (β = .268, 95% CI .012 to .524, P = .041) and perceived neighborhood environment (β = .200, 95% CI .018 to .383, P = .031) but not related to declines in enjoyment motivation, P = .130; social motivation, P = .324; perceived parental support, *P* = .959; perceived friend support, P = .102; or perceived barriers, P = .136. After further adjustment for race, the decline in physical activity was inversely related to decline in perceived barriers (β = -.237, 95% CI -.414 to -.060, P = .009).

### Moderated (i.e., interaction) effects

#### Self-efficacy with perceived parental support

**Fig 3:**
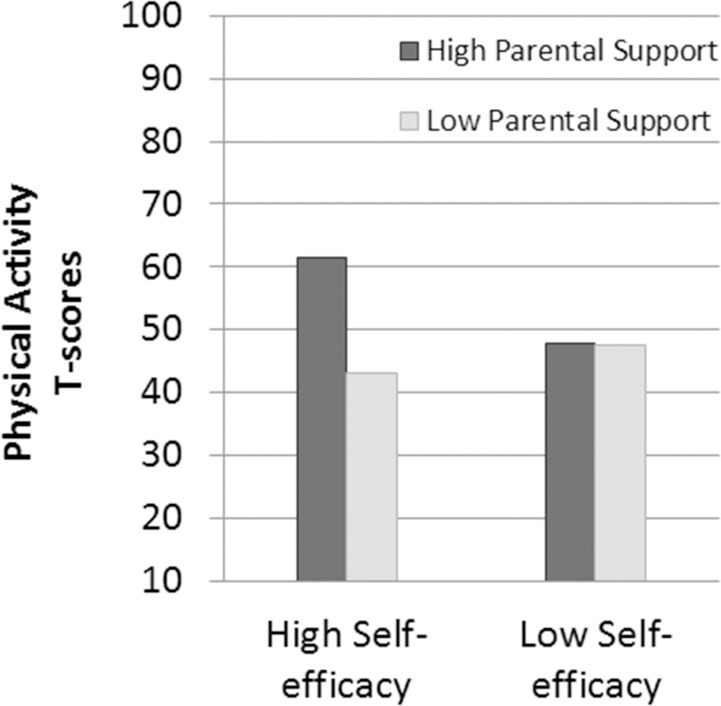
Interaction of change in self-efficacy with change in perceived parental support.

After adjustment for maturity each year and gender, there was an interaction effect between change in self-efficacy and change in perceived parental support on decline in physical activity (z-value = 6.1, p < .001, R^2^ = .54). Physical activity declined least in students who had less of a decline in self-efficacy and less of a decline in parental support compared to other students (Fig 3).

#### Perceived neighborhood environment with perceived barriers

**Fig 4:**
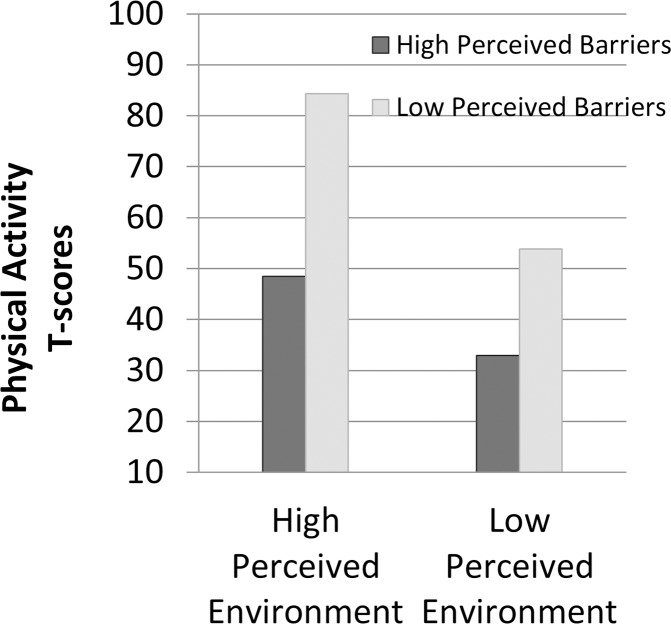
Interaction of change in perceived neighborhood environment with change in perceived barriers.

After adjustment for maturity each year, gender and race, there was an interaction effect between change in the perceived environment and change in perceived barriers on decline in physical activity after further adjustment for race (z-value = 2.21, p = .027, R^2^ = .47). Physical activity declined least in students who maintained more favorable perceptions of their neighborhood environment and had the largest decline in barriers compared to other students (Fig 4). The effect was no longer significant after further adjustment for parental education and poverty (P = .828).

#### Perceived parental support with perceived friend support

**Fig 5:**
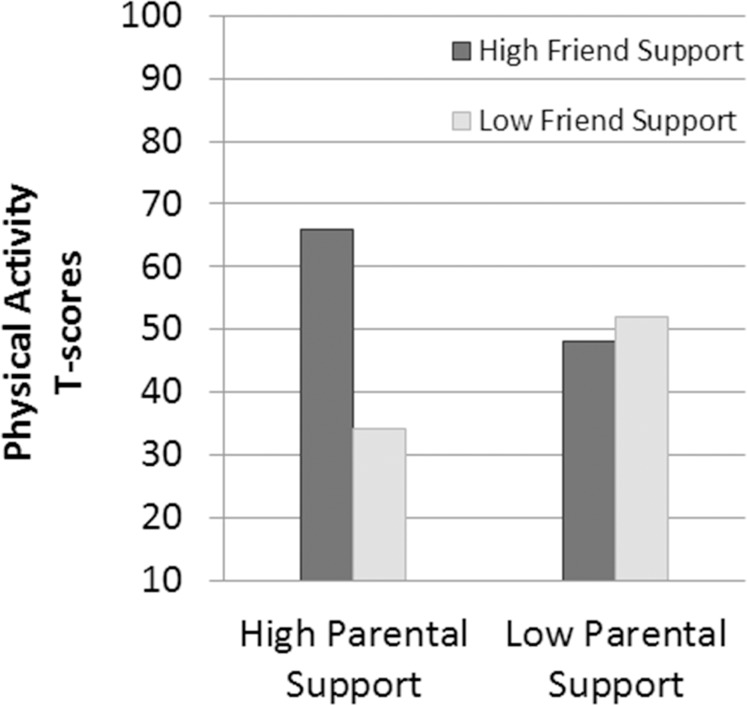
Interaction of change in perceived parental support with change in perceived friend support.

After adjustment for maturity each year and gender, there was an interaction effect between change in perceived parental support and change in perceived friend support on decline in physical activity (z-value = 3.12, p = .002, R^2^ = .53). Physical activity declined least in students who maintained the highest perceptions of both parental and friend support compared to students who had the greatest decline in friend support regardless of parental support (Fig 5).

## Discussion

Naturally-occurring changes in children’s beliefs about barriers to physical activity and their self-efficacy for overcoming them, as well as perceptions of their neighborhood environment and social support from parents and friends, correlated with the age-related decline in children’s physical activity during the transition from childhood to early adolescence. The correlations were direct only for self-efficacy, perceived barriers, and perceived neighborhood environment. Moderating interactions between changes in the variables better accounted for change in physical activity. Physical activity declined least in students who had less of a decline in self-efficacy for overcoming barriers to activity concurrent with less of a decline in perceived parental support. Physical activity also declined least in students who maintained more favorable perceptions of their neighborhood environment and had the largest decline in perceived barriers, as well as less in students who persistently perceived more parental support or friend support for physical activity, compared to those who reported the largest decrease in perceived support from friends. In contrast to our prior finding that enjoyment motivation was related to physical activity in 5^th^ and 6^th^ grades [38], change in enjoyment motivation, like change in social motivation, were unrelated to change in physical activity during the transition from elementary school to middle school.

The average daily decline in total physical activity of ten minutes for boys (16%) and twelve minutes for girls (21%) seen here between 5^th^ and 6^th^ grades is consistent with, though a little less than, the 25% age-related decline in moderate-to-vigorous physical activity observed for boys and girls between ages 11 and 12 years in the National Institute of Child Health and Human Development cohort study [4]. Here and there, the declines could account for a substantial part of the large drop for US children in the rate of sufficient physical activity (from near 50% to just 12%) during their transition to early adolescence [5]. Here, however, interactions between changes in social-cognitive variables (self-efficacy with perceived parental support; perceived neighborhood with perceived barriers; perceived parental support with perceived friend support) had strong effects (approximating 1.5 to 2.5 SD), which accounted for about half the decline in physical activity.

The results are new, because they confirm that changes in these social-cognitive influences on physical activity, which were known to have cross-sectional or predictive associations with physical activity measured mostly by self-report in children and youths [20], are in fact prospectively associated with objectively measured change in physical activity. The results here for self-efficacy and perceived support from parents and friends, based on changes observed across two years, extend prior longitudinal evidence that showed initial levels on similar constructs were predictive of change in physical activity measured by accelerometry among boys and girls across one year from age 9 to 10 years [54]. The findings extend the evidence from our prior studies of modifiable motives (i.e., social and enjoyment) and beliefs (i.e., self-efficacy and perceived barriers, support from parents and friends, and the neighborhood environment) about physical activity among adolescent girls [26–29].

Strengths of the study are the use of an objective measure of physical activity and the repeated observations in a large cohort of boys and girls followed for two years, from the 5th grade through the 7th grade. An additional strength of the study is the application of latent growth modeling, which uses each student’s trajectory of change to estimate the typical change across students in physical activity and the social-cognitive determinants, as well as the variance of those changes, while also adjusting for initial values observed in the 5^th^ grade. This approach permits a fuller test of correlated changes across time than prior longitudinal approaches, which may have failed to detect significant associations among similar variables when analysis was limited to less precise estimates of change [27, 55]. A novel aspect of the growth modeling was the adjustment for maturational differences between boys and girls [21, 25]. The measure of biological maturation was limited to an estimate based on stature, which is nonetheless a preferred method that is practicable in large cohort studies [24, 56].

Novel findings of the study are the moderating effects of concurrent changes in social-cognitive factors, which interacted to influence change in physical activity. Prior observational studies that used a longitudinal design to test social-cognitive theories of adolescent physical activity relied on a self-report of physical activity and did not test whether changes in social-cognitive variables interacted [57]. The absence or presence of main effects on physical activity in those studies might have obscured more complex, moderated (i.e., interactive) effects on physical activity among key social-cognitive variables. Here, for example, changes in perceived support from parents or friends were not directly related to change in physical activity, but change in perceived parental support interacted with changes in self-efficacy and perceived friend support to influence change in physical activity. Less than 20% of about 100 published observational studies on social support by parents or friends of youths’ physical activity used a longitudinal design [58, 59], and just a handful assessed the association of change in support with change in physical activity, measured by self-report or accelerometry [58]. Those studies of change reported varying positive and null effects, but they collectively did not examine whether change in social support had indirect effects on physical activity by mediating or moderating (i.e., interacting with) the effects of other social-cognitive factors [26]. Involving parents in interventions based on social-cognitive theories has typically had little or no effect on children’s physical activity [60], but to our knowledge, tests of moderators of physical activity change after intervention have been limited mainly to stable characteristics of students or their environment not practically amenable to change by intervention [61, 62].

The findings also extend evidence for the longitudinal measurement equivalence/ invariance of the social-cognitive measures, previously validated in 6^th^ and 8^th^ grade girls, to 5^th^ graders and to boys from the 5^th^ to 7^th^ grades. The scales can be tested in other large samples of boys and girls and used to measure change or test the variables’ putative roles as mediators or moderators of physical activity change in interventions during middle school. Results were similar for boys and girls. Findings were not influenced by race/ethnicity, except for the influence on physical activity change by the interaction of perceived environment with perceived barriers. Sample size was not big enough to statistically compare the measurement equivalence of change and relationships with change according to race or ethnic group.

Multi-level intra-class correlations indicated that the variation between schools in both physical activity and the social-cognitive factors was insufficient to test cross-level effects between school features and student variables. Nonetheless, errors of the parameter estimates were properly adjusted to account for nesting of students within schools. The findings are limited to students attending two school districts in South Carolina, so it is not known how well they generalize to other parts of the nation or to similarly aged children educated in private schools or alternative settings such as home-schooling or online education, where social influences on physical activity may differ. Except for extinguishing the moderator effect of the interaction between perceived neighborhood environment and perceived barriers, results were not influenced by socio-economic variables. However, the socio-economic measures were limited to parents’ reports of education level and the percentage of families at or below poverty level based on US census tract data.

The ages studied represent a key period of biological maturation when growth is accelerated. Past studies of the association between biological maturity and physical activity have yielded conflicting results but suggested that the lower physical activity observed among early-maturing girls is explainable by increasing BMI [24]. The results we report here indicate that biological maturity indexed by the growth spurt are inversely related to physical activity and explain higher physical activity among boys in the 5^th^ grade and part of the steeper decline in physical activity among girls from the 5^th^ to the 6^th^ grade, after which most boys have caught up to girls in maturity. In the Avon Longitudinal Study of Parents and Children birth cohort study, maturity (similarly defined as percentage of predicted adult stature) was inversely related to physical activity measured by an accelerometer at age 11 years, but maturity at age 11 did not predict physical activity at age 13 years in either sex [56]. However, the time-varying influence of maturity was not modeled each year in that study, as was done here. Additional longitudinal research is needed to examine how biological maturation influences physical activity [21], as well as whether it determines or modifies youths’ motivation for physical activity within the changing social context of middle school [24, 25].

In conclusion, these findings indicate that modifiable beliefs (i.e., self-efficacy and perceptions of barriers, perceived support from parents and friends, and the perceived neighborhood environment) about physical activity are plausible, interacting targets of interventions designed to mitigate the marked decline in physical activity that occurs during the transition between elementary and middle schools.
